# Beyond AREDS Formulations, What Is Next for Intermediate Age-Related Macular Degeneration (iAMD) Treatment? Potential Benefits of Antioxidant and Anti-inflammatory Apocarotenoids as Neuroprotectors

**DOI:** 10.1155/2020/4984927

**Published:** 2020-12-08

**Authors:** Serge Camelo, Mathilde Latil, Stanislas Veillet, Pierre J. Dilda, René Lafont

**Affiliations:** ^1^Biophytis, Sorbonne Université-BC9, 4 Place Jussieu, 75005 Paris, France; ^2^Sorbonne Université, CNRS-Institut de Biologie Paris Seine (BIOSIPE), 75005 Paris, France

## Abstract

Age-related macular degeneration (AMD) is the commonest cause of severe visual loss and blindness in developed countries among individuals aged 60 and older. AMD slowly progresses from early AMD to intermediate AMD (iAMD) and ultimately late-stage AMD. Late AMD encompasses either neovascular AMD (nAMD) or geographic atrophy (GA). nAMD is defined by choroidal neovascularization (CNV) and hemorrhage in the subretinal space at the level of the macula. This induces a rapid visual impairment caused by the death of photoreceptor cells. Intravitreal injection of anti-vascular endothelial growth factor (VEGF) antibodies is the standard treatment of nAMD but adds to the burden of patient care. GA is characterized by slowly expanding photoreceptor, and retinal pigment epithelium (RPE) degeneration patches progressively leading to blindness. There is currently no therapy to cure GA. Late AMD continues to be an unmet medical need representing a major health problem with millions of patients worldwide. Oxidative stress and inflammation are recognized as some of the main risk factors to developing late AMD. The antioxidant formulation AREDS (Age-Related Eye Disease Studies), contains *β*-carotene, which has been replaced by lutein and zeaxanthin in AREDS2, are given to patients with iAMD but have a limited effect on the incidence of nAMD and GA. Thus, to avoid or slowdown the development of late stages of AMD (nAMD or GA), new therapies targeting iAMD are needed such as crocetin obtained through hydrolysis of crocin, an important component of saffron (*Crocus sativus L*.), and norbixin derived from bixin extracted from *Bixa orellana* seeds. We have shown that these apocarotenoids preserved more effectively RPE cells against apoptosis following blue light exposure in the presence of A2E than lutein and zeaxanthin. In this review, we will discuss the potential use of apocarotenoids to slowdown the progression of iAMD, to reduce the incidence of both forms of late AMD.

## 1. Introduction

Age-related macular degeneration (AMD) is the main cause of blindness in the industrialized world with over 30 million people suffering from this disease [1]. In the US, the number of patients is expected to increase from 9.1 million in 2010 to up to 17.8 million in 2050 [1, 2]. The situation is even more critical in Europe, as it is estimated that by the end of 2020 almost 59 million Europeans will develop at least one form of the disease [3]. Novel therapeutic strategies are needed to reduce disease prevalence [1, 2, 4]. AMD is a chronic disease that may progress slowly from early AMD to intermediate AMD (iAMD) and ultimately late-stage AMD, either neovascular (nAMD) or geographic atrophy (GA). Although visual acuity under photopic conditions remains good in the early stages of AMD, disease impact on patients with iAMD is severe with a loss in quality of life owing to poor visual acuity under low luminance conditions that affects many aspects of normal life activities [5–8]. Moreover, the economic burden of AMD on society is very high and will increase as the population ages [9, 10].

Neovascular AMD is defined by choroidal neovascularization (CNV) and hemorrhage in the subretinal space at the level of the macula. This induces the rapid death of photoreceptor cells and then rapid loss of vision [11]. Currently, three drugs targeting vascular endothelial growth factor (anti-VEGF) are in use for nAMD. Two of them, ranibizumab (Lucentis®, Genentech) and aflibercept (Eylea®, Regeneron), are approved for this indication. The third one, bevacizumab (Avastin®, Genentech), is used off-label for nAMD and is a cheaper and then a more cost-effective treatment [12]. The efficacy of the three treatments administered via monthly or as-needed intravitreous injections has been shown in numerous studies [13]. However, undetected occult CNV and/or incomplete compliance of patients omitting some injections lead to suboptimal treatment efficacy [12]. Moreover, development of macular atrophy or fibrosis secondary to nAMD jeopardizes long-term visual acuity in these patients [14, 15]. To improve treatment efficacy, quality of life, and compliance of patients with wet AMD, long-term antiangiogenic therapies or combination therapies are currently in development by several companies [13]. In addition, based on the results of phase III clinical trials (HAWK and HARRIER), brolucizumab (Novartis) has received market authorization in the US and Europe under the name Beovu® in the fall of 2019. Beovu® offers both greater fluid resolution versus Eylea® and the ability to maintain eligible nAMD patients on a three-month dosing interval immediately after a three-month loading phase [16].

GA is characterized by slowly expanding lesions of photoreceptors and retinal pigment epithelium (RPE) leading to progressive retinal degeneration and dysfunction [17–19]. Severe and irreversible loss of central vision may result from GA when the macula is involved. The recent results of several clinical trials testing anticomplement strategies showed a reduction of GA growth rate. The FILLY, NCT02503332 testing Pegcetacoplan® (APL-2) from Apellis Pharmaceuticals Inc., [20] and NCT02686658 using Zimura® from Iveric Bio reported approximately a reduction by 30% of the growth of GA. Another clinical trial (BEACON, NCT02087085) testing brimonidine (Brimo DDS®), a neuroprotective molecule developed by Allergan and now owned by AbbVie [21], reduced GA growth by approximately 12%. These results await confirmations by phase III clinical trials, and new therapeutic options will not be available to patients until the respective drug candidates are registered by US and European authorities. It should also be noted that the extension of areas of GA was not completely halted, and no visual acuity (VA) gain was reported. Thus, even if these drugs become commercially available, it is expected that GA will remain a major unmet clinical need [17, 22]. Therefore, the development of new drugs or alternative strategies able to entirely stop GA progression is still required. Multiple factors have been implicated in the evolution of iAMD and the development of both late forms of AMD. These include age [23, 24] and environmental factors (mainly smoking) [25, 26]. Genetic factors are also involved in the pathology, the main one being polymorphism in the factor H of complement (CfH), which increases by 3- to 6-fold the risk of developing AMD [27–30]. Other genetic polymorphisms have been associated with increased AMD risk [2], including polymorphisms in the ARMS2/HTRA1 loci [31, 32]. ARMS2 polymorphism affects the function of retinal mitochondria, while HTRA1 regulates transforming growth factor-*β* expression involved in angiogenesis and extracellular matrix deposition. A pivotal role for inflammation in iAMD and both forms of late AMD has also been reported [33, 34]. The initial cause of inflammation and the subsequent retinal destruction observed during AMD remain a subject of debate [35]. It is most probably caused by oxidative stress, which is recognized as a major risk factor in AMD development [36–43]. Therefore, antioxidant and immunosuppressive therapies are likely to be beneficial for patients with iAMD and may reduce the incidence of GA and nAMD. Here, we review existing knowledge on iAMD physiopathology and treatment modalities and propose that apocarotenoids, thanks to their very high antioxidant activity and anti-inflammatory properties, could benefit patients with iAMD and potentially reduce the incidence of late AMD.

## 2. Material and Methods

Although not a systematic review, relevant studies published and available on PubMed up to the 7th of July 2020 were searched for. Using Boolean operators (e.g., AND, OR), the applied search terms included combinations of the following key words: “AREDS”, “AREDS2”, “lutein”, “zeaxanthin”, “saffron”, “crocetin”, “crocin”, “bixin”, “norbixin”, “annatto”, “ocular”, “retina”, “macula”, “macular”, “age-related macular degeneration”, “AMD”, “intermediate AMD”, “(anti)-inflammatory”, and “(anti)-oxidant”. To minimise the risk of omitting relevant studies, the reference lists of all eligible papers were also manually checked. Only publications in the English language were included. In addition, although nonexhaustive, we searched for formulations of supplements containing carotenoids and available for purchase on the web.

## 3. Results and Discussion

### 3.1. Intermediate AMD

**Figure 1:**
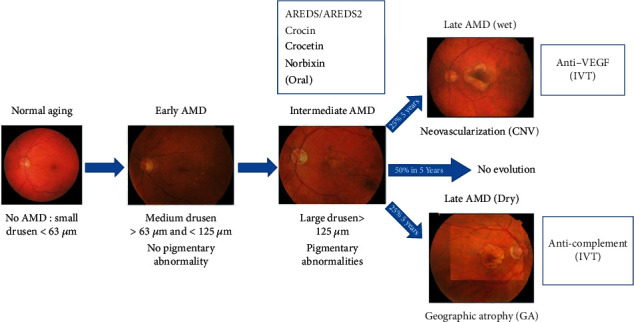
Summary of AMD clinical evolution adapted from the Beckmann classification system [42] and treatment modalities. AMD: age-related macular degeneration; CNV: choroidal neovascularization; GA: geographic atrophy; IVT: intravitreal injections; VEGF: vascular endothelial growth factor.

IAMD can evolve towards the fast-developing exudative form or the atrophic form of AMD or some combination of these two endpoints (Figure 1) [44]. IAMD is characterized by the progressive accumulations of lipid and protein waste between the Bruch's membrane and the basal side of the RPE and called drusen. In many patients with iAMD, waste materials also accumulate and form Reticular Pseudo-Drusen (RPD) between the apical side of the RPE and the photoreceptor outer segment [45–48]. Rod photoreceptors mediate “night vision” (scotopic visual process), whereas diurnal (photopic) vision is mediated by cones [45]. The first visual signs of iAMD are poor night vision associated with the disappearance of rod photoreceptors [45, 46, 48]. Visual tests used to follow the declining visual functions during iAMD include dark adaptation (DA), scotopic and/or mesopic microperimetry, and low-luminance visual acuity (LLVA) [49]. In addition, changes in multifocal electroretinogram (mfERG) response density and latency [50] and retinal flicker sensitivity [51] are also used to follow loss of vision in patients with mild to moderate AMD [52]. Rod-related visual function depends upon unimpaired transportation of nutrients and in particular vitamin A (the precursor of retinal, a key component of the visual pigment rhodopsin) from the choroidal vasculature through RPE cells up to the rod photoreceptors. It has been proposed that drusen impairs this transport. In addition, RPD might also disturb the close interaction between RPE cells and rods that is necessary for the visual cycle to occur. It is therefore not surprising that drusen and RPD are a major risk factor for apparition of early visual deficits [53] and for evolution of iAMD to late stage AMD (nAMD or GA) [54–58]. Strategies attempting to slowdown the evolution of iAMD towards late stages of the disease appear to be an interesting option. At present, most medications for patients with intermediate stage of AMD rely on dietary supplements based on the Age-Related Eye Disease Studies: AREDS and AREDS2 (Table 1).

### 3.2. AREDS/AREDS2 Formulations

**Figure 2:**
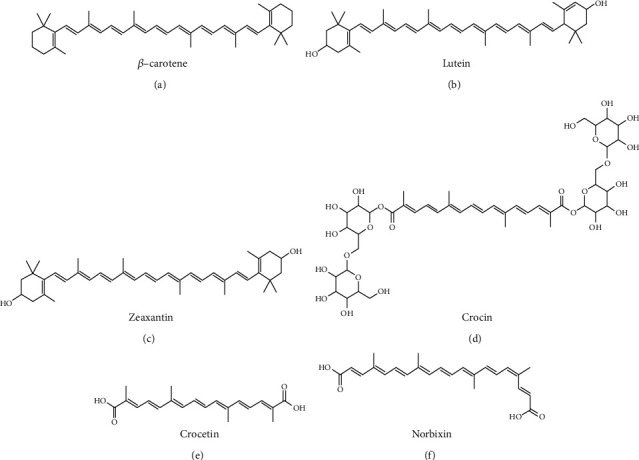
The chemical structures of antioxidant molecules that are used or could be used for the treatment of the intermediate form of AMD.

The AREDS formulation was developed empirically [59]. The effects of the antioxidant AREDS formulation were analyzed in a blinded, randomized, and controlled study on several thousand patients. The original AREDS formulation (described in Table 1) contained *β*-carotene (Figure 2(a)) and vitamin C, vitamin E, and zinc among other components. Supplementation with AREDS reduced the risk of developing late AMD by an estimated 25% (5 years incidence of late AMD decreased from 28% to 20%) [59]. However, treatment with AREDS over 8 years did not entirely block iAMD progression, and a loss of vision still occurred in patients. Moreover, *β*-carotene was reported to increase the risk of developing lung cancer in cigarette smokers [60]. Since then, *β*-carotene has been replaced by the macular xanthophyll lutein (Figure 2(b)) and zeaxanthin (Figure 2(c)) in the AREDS2 formulation (Table 1) [61]. AREDS2 supplementation appeared superior to the AREDS formulation to reduce the risk of developing late AMD [61]. Nevertheless, subsequent meta-analysis showed that the benefits of antioxidants and of the AREDS/AREDS2 antioxidative formulations were limited [4, 60]. Thus, the development of an improved oral and safe treatment with better efficacy on iAMD evolution is still needed and is the focus of intensive current research.

Targeting oxidative stress is the rationale behind the AREDS/AREDS2 protocols. *β*-carotene, lutein, and zeaxanthin (L/Z) are known for their antioxidant properties. In the organism of mammals, carotenoids originate exclusively from the diet [62, 63]. *β*-Carotene is rapidly cleaved into retinol/vitamin A in the liver. Thus, ingested *β*-carotene is not found in the eye [62, 63]. However, *β*-carotene exerts some protective effects in AREDS supplemented patients, suggesting that these effects are systemic. By contrast to *β*-carotene, oxidized carotenoids including xanthophylls are not cleaved in the liver. The xanthophylls (L/Z), which can be extracted from marigold flowers (*Tagetes erecta* L.), are naturally present in the mammalian retina and most particularly at the level of the macula and fovea [62, 63]. As a result, L/Z are macular pigments. The protective effects of L/Z appear to be both systemic and local. The local action of L/Z, partly depending on their ability to filter phototoxic blue light radiation due to their maximum absorption around 460 nm and via their antioxidant activity, has been demonstrated [64]. Supplementation with L/Z augments their intraocular concentrations in animals [65]. Epidemiological evidence has shown that patients with lower concentrations of macular pigment optical density (MPOD) measurements are at a higher risk of developing AMD. It was suggested that the MPOD concentrations depend on the amount of these products in the diet and that ocular concentrations of L/Z would be increased in a small cohort of patients from an AREDS2 ancillary study receiving L/Z supplementation [66]. However, MPOD was not modified after 6 months of lutein and zeaxanthin dietary supplementation [67]. By contrast and similarly to *β*-carotene, L/Z protective effects are also linked to their systemic antioxidant properties. Indeed, daily supplementation of L/Z in rats for 42 days significantly increased the serum levels of catalase, an antioxidant enzyme, compared to serum concentrations in the nonsupplemented rats. Simultaneously, the total antioxidant capacity was increased significantly by L/Z supplementation over placebo, indicating that L/Z supplementation has a profound action on the systemic antioxidant defense system [64]. Accordingly, the serum concentration of L/Z was doubled in patients receiving the AREDS2 formulation for several years compared to the normal population without supplementation [61]. Additional components such as zinc, a cofactor of superoxide dismutase (SOD; a key antioxidant enzyme), most probably amplify the systemic antioxidant effects of the AREDS2 formulation (Table 1). At the systemic and local levels, L/Z act on several types of cells important for AMD physiopathology. *In vitro*, lutein alone reduces the VEGF expression in RPE cells [68] and also reduced expression of interleukin- (IL-) 6, VEGF, and matrix metalloproteinase- (MMP-) 9 in macrophages, which have been implicated in AMD [68, 69]. Similarly, production of the chemokine (C-C motif) ligand 2 (CCL2) by microvascular endothelial cells and RPE was downregulated by lutein *in vitro* [68]. Lutein also activates nuclear factor erythroid 2-related factor 2 (Nrf2), the master gene of antioxidant response, in ARPE-19 cells, a RPE cell line, *in vitro* [70]. Moreover, antiangiogenic and anti-inflammatory effects were also observed *in vivo* in the model of choroidal angiogenesis following laser-induced CNV in mice treated with lutein showing reduced infiltration by macrophages, reduced production of inflammatory cytokines (IL-1-*β*, IL-12, and TNF-*α*) and chemokines (including CCL2, CCL3, and CCL5), and limited neovascularization at the site of laser impact [68, 71]. These effects are linked to reduced NF-*κ*B activation, due to inhibition of I*κ*B-*α* degradation [68]. Because oxidative stress and inflammation have been implicated in AMD pathophysiology, these observations probably explain the reduced risk of AREDS2-treated patients to develop late AMD and particularly nAMD [61]. In addition, *in vivo* supplementation of L/Z is also protective in two animal models of retinal degeneration, which develop a phenotype similar to the atrophy observed in patients' eyes with GA. In aged CCL2/CX3CR1-deficient mice on a Crb1^rd8^ genetic background, L/Z supplementation reduced ocular accumulation of N-retinylidene-N-retinylethanolamine (A2E, a major toxic component of drusen) [72]. L/Z also inhibited retinal IL-1*β*, TNF-*α*, Cox2, iNOS, and VEGF expression *in vivo* and preserved the retinal architecture [72]. In the light-challenged albino Balb/c mice model of retinopathy, supplementation with L/Z reduced the expression of several endoplasmic reticulum and oxidative stress markers and a lower number of apoptotic photoreceptors [73]. These effects correlated with preserved retinal structures and functions measured by electroretinography (ERG) [73]. Finally, in an *in vivo* model of oxidative stress following consumption of a high fat diet, oxidative damage, and inflammation cascade was partially reversed by supplementation with L/Z, and this effect involved Nrf2 regulation [74]. However, as said above, despite these convincing proofs of efficacy *in vitro* and *in vivo*, interest in AREDS supplementation for humans appears limited. Indeed, despite a reduction of the incidence of formation of large drusen (>125 mm) and pigmentary abnormalities has been reported, no effects were observed on overall incidence of iAMD following intakes of pro-vitamin A carotenoids and dietary vitamin E [75] or L/Z intake [76]. In another study, an effect on early AMD incidence was only observed in women younger than 75 years old only [77], but nonsignificant effects on incidence of late AMD were reported [76, 77]. Thus, the use of other compounds with more potent antioxidative properties could improve the management of iAMD.

### 3.3. Crocins and Crocetin

Powder of saffron (*Crocus sativus* L., Iridaceae) has been used in traditional medicine since antiquity. Between 100,000 and 200,000 saffron flowers are required to produce 1 kg of dry powder. Saffron contains safranal and a mix of several antioxidant molecules derived from *β*-carotene including crocin and crocetin. This review focuses on the last two compounds. Crocin and crocetin are also found in larger amounts in the fruits of gardenia (*Gardenia jasminoides* Ellis). Crocetin is a dicarboxylic 9,9′-diapo-carotenoid (C_20_H_24_O_4_) (Figure 2(d)) derived from the naturally occurring crocin, its digentiobioside (Figure 2(e)) [78]. Crocetin and the various isoforms of crocin (crocins) are bioavailable following oral ingestion and are present in the blood plasma in the form of native crocetin and mono- and di-glucuronide crocetin conjugates [79], all having antioxidative properties. Crocetin and crocins also have the capacity to significantly absorb light at 256, 315, 423, and 449 nm and at 235, 324, 432, and 457 nm, respectively [80]. Based on these properties, saffron components, crocins, and crocetin could be beneficial for iAMD patients. Several *in vitro* and *in vivo* studies testing the efficacy of saffron, crocins, or crocetin in models reproducing pathophysiological process of iAMD have been performed and are summarized hereafter. *In vitro*, saffron extracts and its major components display neuroprotective actions through several mechanisms. For instance, crocin protects bovine and nonhuman primate photoreceptors against cell death induced through strong intensity illumination with blue or white light [81]. Preventive protection by crocin is dose-dependent (EC_50_ = 30 *μ*M) and is associated with inhibition of caspase activity [82]. Similarly, it has been shown that saffron partially preserves the viability of mouse primary retinal cells and a photoreceptor cell line (661 W cells) exposed to toxic doses of ATP. In this model, neuroprotection by saffron was associated with a reduction of intracellular calcium increase induced by ATP and was mediated through saffron action on the purinergic P2X7 receptor [83]. In parallel, it has been shown *in vitro* as well that crocetin reduces the effects of oxidative stress induced by *tert*-butyl-hydroperoxide in the ARPE-19 cell line [84]. Pretreatment of RPE cells with crocetin prevented apoptosis evidenced by lactate dehydrogenase release, intracellular ATP depletion, and nuclear condensation [84]. Crocetin preserved junctional and cytoskeleton integrity that are essential for RPE functionality [84]. The neuroprotective effect of saffron components could also be related to their known anti-inflammatory properties. Retinal microglial cells play critical roles in maintaining retinal homeostasis and ocular immune and inflammatory responses. During AMD, chronic microglial activation has been implicated in neuronal degeneration through the release of proinflammatory cytokines and neurotoxic factors. The *in vitro* effects of crocin and crocetin on proinflammatory gene expression in activated BV-2 microglia cell line and primary microglia were examined [85]. Both crocin and crocetin were shown to inhibit lipo-polysaccharides- (LPS-) induced NF-*κ*B activation, tumor necrosis factor-*α* (TNF-*α*), IL-1*β*, nitric oxide (NO), and reactive oxygen species (ROS) production by rat microglial cells [85]. Interestingly, amyloid-*β* accumulates in drusen in the eyes of patients with AMD, and crocin has been shown to reduce NO release from microglia stimulated with interferon-*γ* and amyloid-*β* [85]. In a similar study, crocins stimulated microglial phagocytosis, which is important for retinal homeostasis, and significantly reduced gene expression of IL-6 and CCL2 [86]. These authors also reported that crocin inhibited iNOS gene expression and NO production in LPS-challenged BV-2 microglia [86]. These results suggest that crocins and crocetin may provide neuroprotection by reducing the production of various neurotoxic molecules by activated microglia. Accordingly, *in vivo* microglial activation in retinas of albino rats exposed to light damage was reduced by saffron treatment [87]. In the same experiments, Di Marco and coworkers reported that saffron inhibited the MMP3 expression and activity, which was associated with improved retinal structure [87]. These animal studies, as well as others, confirm the neuroprotective effects observed following saffron treatment. Indeed, oral supplementation for 20 weeks with saffron in the model of apoE^−/−^ mice fed with a high-fat diet resulted in preservation of retinal thickness when compared with non-supplemented mice [88]. The outcomes of the study suggest the potential neuroprotective role of saffron against retinal damage induced by oxidative stress. Moreover, supplementation for 6 weeks with 1 mg/kg/day of *β*-carotene or saffron preserved retinal histology of 2-month-old albino rats exposed during 24 h to intense light (1000 lux) [89]. Interestingly, saffron was more effective than *β*-carotene to preserve photoreceptor functionality tested through electroretinography (ERG). This suggested that saffron administration could preserve visual function in iAMD patients and perhaps more efficiently than the *β*-carotene-containing AREDS formulation. The effects of saffron supplementation in humans with various ocular disorders, including iAMD, have been reviewed recently [90, 91]. In 2010, Falsini and coworkers reported a significant improvement of visual function determined through measure of multifocal ERG (mfERG) in patients with iAMD taking orally 20 mg daily of saffron (representing 0.6 mg of crocetin) per day for three months [92]. A subsequent study of the effects of long-term oral administration of saffron over a period of 12 months by the same authors [93] reported sustained improvement of mean fERG sensitivity and mean visual acuity in patients with early AMD. More recently, the efficacy and safety of three-month oral saffron supplementation was assessed in a randomized, double-blinded, and placebo-controlled crossover trial on 100 adults with mild/moderate AMD. Unfortunately, saffron supplementation only moderately improved mean visual acuity and mfERG, including in participants with AMD using AREDS supplements concomitantly [50]. Nevertheless, a comparison of long-term (29 months) supplementation with saffron versus L/Z has shown that visual function (measured by mfERG) remained stable in the saffron-treated group while it had deteriorated in the L/Z-treated group [87] supporting the benefit of saffron compared to the AREDS2 formulation. This also suggests that other compounds with a higher antioxidative potential than crocin and crocetin could potentially be even more beneficial to visual function in the early stages of AMD. In recent years, some dietary supplements containing crocins/crocetin in addition to L/Z have appeared on the market (Table 1), but their use is limited by the lack of convincing clinical trials data and the preponderant use of AREDS/AREDS2 formulations.

### 3.4. Norbixin

9′-*cis*-Norbixin is a 6,6′-di-*apo*-carotenoid extracted from annatto (*Bixa orellana*) seeds. Norbixin (Figure 2(f)) structure is close to that of crocetin. Norbixin is used as a food colouring agent (E160b) [94]. Tolerability of norbixin is well known, based on both animal and human studies, and the safety data support the use of norbixin as a food additive (with an acceptable daily intake of 0.3 mg norbixin/kg of body weight per day). It has been demonstrated in cellular and animal models that norbixin limits the appearance of symptoms similar to those observed during iAMD in humans [95]. *In vitro*, norbixin preserved the survival of primary cultures of porcine RPE cells challenged with A2E in the presence of blue light illumination [95]. Interestingly, it was shown that the effectiveness of norbixin in this *in vitro* test was superior to the photo-protective effects of L/Z and crocetin [95]. However, norbixin and crocetin effectiveness *in vitro* and *in vivo* against endoplasmic reticulum stress is similar [96]. *In vivo*, acute norbixin treatments of albino Wistar rats and ABCA4^−/−^/Rdh8^−/−^ double-knockout mice exposed to intense light (Blue Light Damage (BLD) model) protect retinal tissues and photoreceptor cells [95]. Similar results were observed in albino Balb/c mice exposed to BLD [96]. In addition, supplementation of ABCA4^−/−^/Rdh8^−/−^ mice with a diet containing norbixin also prevented the reduction of rod and cone photoreceptor electrical activity measured by scotopic and photopic ERG amplitude, respectively [95, 92]. This indicates that norbixin could potentially preserve “night” and “day” visual acuity in humans. Interestingly, norbixin reduced the uptake of A2E by porcine RPE cells *in vitro* [95]. A2E accumulation is observed during the early stages of AMD. Accordingly, long-term *in vivo* administration of norbixin reduces the ocular accumulation of A2E in ABCA4^−/−^ and Rdh8^−/−^ mice [97], suggesting that norbixin could slowdown the subretinal accumulation of A2E that is observed during iAMD. It was further demonstrated *in vitro* that norbixin significantly reduced the production of VEGF and of several inflammatory cytokines, such as IL-6 and IL-8, in porcine RPE cells cultivated in the presence of A2E (V. Fontaine, M. Fournié, E. Monteiro, T. Boumedine, C. Balducci, L. Guibout, M. Latil, PJ. Dilda, J.-A. Sahel, S. Veillet, R. Lafont, S. Camelo, manuscript posted on bioRxiv) [98]. These *in vitro* data confirm the anti-inflammatory effects of norbixin observed in human studies *in vivo* [99, 100]. *In vivo*, consumption of a high-fat meal induces the production of the proinflammatory cytokines IL-1, TNF-*α*, and IL-6 in human plasma, which can be reduced by norbixin treatment [94]. Interestingly, norbixin also reduces ROS production by ARPE-19 following exposure to antimycin A-induced oxidative stress [101]. Conversely, in human plasma, norbixin elevated the levels of the antioxidant glutathione and of glutathione peroxidase *in vivo* [100]. In addition, oral norbixin administration to humans following a high-fat meal reduced the plasma concentration of malondialdehyde (MDA) [100]. These observations are promising, since MDA level increases have been observed during early stages of AMD [102]. Altogether, this suggests that norbixin, through its antioxidative and anti-inflammatory activity, may be beneficial to treat patients with iAMD. Moreover, norbixin through inhibition of A2E accumulation could slow or even stop the progression from iAMD to late dry AMD and could preserve normal rod-mediated “night vision” in iAMD patients. The safety and efficacy of norbixin or related molecules should be evaluated in future clinical trials in patients with iAMD. However, drug development using norbixin and apocarotenoids in general, as active principles, requires a better understanding of their exact mode of action. We recently started to explore the molecular clues explaining the antioxidant and anti-inflammatory properties of norbixin and found out that it modulates very precisely the activity of certain nuclear receptors [98, 101]. Due to the pleiotropic effects of nuclear receptors that have been implicated in AMD [103], this observation supports the broad beneficial effects of norbixin.

## 4. Conclusion

There is no effective therapeutic strategy for the late form of dry AMD, and intraocular treatments of nAMD are costly and do not prevent long-term loss of vision. As both GA and nAMD originate from iAMD, treating patients at this early stage of the disease could potentially prevent the development of late AMD. Since oxidative stress and inflammation appear to play an essential role in iAMD, the use of therapeutic strategies is aimed at reducing oxidative stress, and inflammation is potentially attractive. At present, prescribing the antioxidant AREDS/AREDS formulations to iAMD patients is the only available therapeutic strategy to reduce late AMD incidence, but its effectiveness appears limited. Here, we described the potential use of apocarotenoids such as crocin, crocetin, and norbixin, for long-term therapy to slowdown the progression of iAMD towards late stages of the disease. Developing such new and more effective treatments for patients with iAMD could drastically reduce the incidence of late forms of AMD in the general population and then could reduce the burden for society as a whole. Indeed, limiting the development of GA and nAMD will not only benefit patients by improving their quality of life but also provide “peace of mind” to the healthy and caregivers. It is also expected that development of such oral drugs will reduce costs to healthcare providers if it becomes the preferred treatment of patients with the intermediate form of AMD.

## Figures and Tables

**Table 1:** Carotenoid content of various preparations as announced in the commercial advertisement (but not verified).

Product	Supplier	Carotenoid amount per day
AREDS1 original formula	—	B15
AREDS2 original formula	—	L 10, Z 2
AREDS2 Preser vision	Bausch & Lomb	L 10, Z 2
AREDS2 plus Zn free	Eyepromise	L 10, Z 10
AREDS2 with resveratrol	Fortifeye vitamins	L 10, Z 2, A 2
Advanced AREDS2 formula	Vitalux	L 10, Z 2
AREDS2	VitOptics	L 10, Z 2
Macula-Z	Horus Pharma	L 10, Z 2
Nutrof	Thea Pharma	L 10, Z 2
Vitalux plus	Alcon/Novartis	L 6, Z
Lutein+zeaxanthin	Piping rock	L 20, Z 1
Senior vision care complex	Piping rock	L 5, Z 8 *μ*g
Ocuvite	Bausch & Lomb	L 5, Z 1
Suveal duo caps	Densmore Laboratoire	L 10, Z 2
Premium MariLut®	Time Sheet	L 10, M 10, Z 2
MacuGuard	Life Extension	L 10, M/Z 4, C
True vision	Nature City	L 10, M/Z 2, C 0.6
Eye protector	Pure Synergy	L 10, M/Z 5, A 2, C 3
Luteine crocine 20 mg	Essence pure	L 20, Z 2, C 0.6
AffronEye®	Pharmactive	C 0.6

A: astaxanthin; B: beta-carotene; C: crocin; L: lutein; M: *meso*-zeaxanthin; Z: zeaxanthin. Unless otherwise indicated, amounts are in mg.
